# Anti-osteoarthritic effects of ChondroT in a rat model of collagenase-induced osteoarthritis

**DOI:** 10.1186/s12906-018-2149-1

**Published:** 2018-04-19

**Authors:** Jiwon Jeong, Kiljoon Bae, Sun-Gil Kim, Dongwook Kwak, Young-Joo Moon, Chan-Hun Choi, Young-Ran Kim, Chang-Su Na, Seon-Jong Kim

**Affiliations:** 1 Department of Rehabilitation Medicine of Korean Medicine, Mokpo Korean Hospital of Dongshin University, 313 Baengnyeon-daero, Mokpo-si, 58665 Republic of Korea; 2 College of Korean Medicine, Dongshin University, 185 Geonjae-ro, Naju-si, Jeollanam-do 58245 Republic of Korea; 3 Training Department of Bucheon Jaseng hospital of korean medicine, 17 Buil-ro, 191beon-gil, Bucheon-si, Gyeonggi-do 145598 Republic of Korea; 4 College of Pharmacy and Research Institute of Drug Development, Chonnam National University, 77 Yongbong-ro, Buk-gu, Gwangju-si, 61186 Republic of Korea

**Keywords:** Osteoarthritis, Collagenase, ChondroT, Inflammatory cytokine

## Abstract

**Background:**

Previously, we reported that ChondorT showed significant anti-arthritis and anti-inflammatory effects. ChondroT, a new herbal medication, consists of the water extracts of *Osterici Radix, Lonicerae Folium, Angelicae Gigantis Radix, Clematidis Radix, and Phellodendri Cortex* (6:4:4:4:3). The objective of this study was to investigate the effects of ChondroT in collagenase-induced osteoarthritis rat model.

**Methods:**

Osteoarthritis was induced by the injection of collagenase into the right knee joint cavity of rats. The samples were divided into seven groups [intact (*n* = 6), control (*n* = 6), indomethacin (*n* = 6), Joins tab (*n* = 6), ChondroT50 (*n* = 6), ChondroT100 (*n* = 6), and ChondroT200 (*n* = 6)]. The control group was administered normal saline, indomethacin group was administered indomethacin (2 mg/kg), and Joins tab group was administered Joins Tab (20 mg/kg). The ChondroT50, ChondroT100, and ChondroT200 groups were administered 50, 100, and 200 mg/kg of ChondroT, respectively. All oral administrations were initiated 7 days after the induction of arthritis and were continued for a total of 12 days. At the end of the experiment, serum aminotransferase, albumin, blood urea nitrogen, creatinine, leukocyte, and inflammatory cytokines [tumor necrosis factor (TNF)-α, interleukin (IL)-1β, and IL-6] were analyzed. Hematoxylin and eosin (H&E) and safranin O-fast green staining of the articular structures of the knee joint were performed.

**Results:**

TNF-α and IL-1β decreased in the ChondroT100 and ChondroT200 groups compared with those in the control group. IL-6 and aspartate aminotransferase decreased in the ChondroT50, ChondroT100, and ChondroT200 groups compared with that in the control group. Albumin, WBC and lymphocytes decreased in the ChondroT100 and ChondroT200 groups compared with those in the control group. In H&E stain, synoviocytes, cartilage lacunae, and chondrocytes were well preserved in the ChondroT100 and ChondroT200 groups, and safranin O-fast staining showed a clear reaction of proteoglycans in the ChondroT100 and ChondroT200 groups.

**Conclusions:**

Based on these results, it can be proposed that ChondroT has anti-osteoarthritic effects on collagenase-induced rat model.

## Background

Osteoarthritis (OA), known to be associated with a number of clinical symptoms, has become a significant problem worldwide, due to an increase in the aging population [1]. It results in cartilage degradation which, in turn, leads to cartilage bone damage [2]. This cartilage degradation has been recognized to be induced by inflammatory cytokines, such as IL-6, IL-1β, and TNF-α [3, 4]. Upon mechanical stimulation, the cartilage matrix exhibits changes such that the IL-1 and TNF-α are destroyed and reformed by cytoplasmic decomposing enzymes [5, 6]. This process results in the occurrence of joint ankylosis, progressive motor disturbance, pain near joints, tumentia, and redness [7, 8].

Ganghwaljetongyeum (GHJTY) is a traditional Korean herbal medicine commonly used to treat joint pain, limitation of motion, fever, and swelling and to inhibit inflammatory processes associated with arthritis [9]. Our previous study showed that GHJTY which is a complex herbal decoction composed of 18 plants may be effective in attenuating rheumatoid arthritis by inhibiting the production of pro-inflammatory mediators and proliferation of synoviocytes [10].

In order to improve the efficacy and convenience of pharmaceutical prescription, a previous study used bioinformatics to identify the five medicinal herbs with the greatest potential [11]. The five herbs were then combined in a 6:4:4:4:3 ratio, and the water extract solution was named ChondroT, a new complex herbal medication.

ChondroT exhibited a chondroprotective effect and demonstrated multi-target mechanisms related to inflammation and arthritis [12]. In addition, the suppressive effect was greater than that exhibited by GHJTY, suggesting that it had therapeutic potential for the treatment of arthritis [12]. Moreover, it was also found to be effective in treating a rat model of rheumatoid arthritis(RA), where it significantly suppressed the progression of complete Freund’s adjuvant-induced arthritis, evident from a decrease in paw and knee joint swelling. It was also effective in preventing articular cartilage and synovial tissue degeneration [13].

Injecting collagenase into the glenoid cavity is a method commonly used to induce arthritis in rats [14]. It damages the ligament and meniscus and decreases stability, forcing the mechanical stress to affect the synovial cell and cartilage cell metabolism, thereby producing symptoms similar to osteoarthritis [15]. For this reason, collagenase induced rat model is typically used to investigate therapeutic agents with anti-osteoarthritic potential [16].

This was done by analyzing the inflammatory cytokines (TNF-α, IL-1β, and IL-6), performing hematoxylin & eosin (H&E) staining and safranin O-fast green staining of the articular structures of the knee joint to investigate the effects of ChondroT in collagenase-induced osteoarthritis rat model.

## Methods

### Animals

Adult male Sprague–Dawley rats weighing 170–180 g were housed in a room with constant temperature (24–26 °C) and humidity (40%–60%). Food (Pellet, GMO, Korea) and water were available ad libitum. Animals were acclimated to the laboratory environment for 1 week before commencement of the experiment, and all procedures were approved by the Institutional Animal Care and Use Committee of the Dongshin University (DSU-2015-1003-01). After the experiment, all the rats were euthanized using Carbon dioxide (CO2) in accordance with AVMA guidelines.

### Collagenase-induced arthritis and oral Administration of Drug

Arthritis was induced by dissolving 1.5 KU of Collagenase (Type VII: Sigma, USA) in 600 μL of 0.9% sterile saline followed by injection into the left hind knee joint. Forty-two rats were randomly divided into seven groups (*n* = 6) as follows: intact, control, three ChondroT (50, 100, or 200 mg/kg), and two positive control groups (indomethacin 2 mg/kg, Joins Tab. 20 mg/kg). Oral administration of ChondroT was initiated on the 7th day after arthritis induction, and was continued for 12 days thereafter. Animals were anesthetized using 2.5% isoflurane.

### Preparation of herbal materials

The five herbal medicines constituting ChondroT were purchased from Omniherb Co. (Yeongcheon, Korea), and their origin was taxonomically confirmed by Professor Jong-Kil Jeong at the Department of Herbology, College of Oriental Medicine, Dongshin University.

The five herbs (OK, LJ, AG, CM, and PA) were combined in a 6:4:4:4:3 ratio (Table 1). ChondroT was prepared by carrying out water extraction once, using 10-fold solvent at 100 °C for 3 h, and then filtering it (180 mesh). The water extract solution was concentrated using a continuous vacuum evaporator (approximately 55–60 °C, 670 mmHg), and this was followed by vacuum drying using a vacuum drier (720 mmHg) for 8 h. The yield was approximately 29.4%. Voucher specimens (No. GHJTY 501) of the collected herb samples were deposited in the herbarium of Jung Woo Shin Yak (Asan, Korea).

**Table 1:** Composition of ChondroT

Latin name	Scientific name	Family	Rate	Source
Osterici Radix	*Ostericum koreanum* Maximowicz	Umbelliferae	6	Korea
Angelicae Gigantis Radix	*Angelica gigas* Nakai	Umbelliferae	4	Korea
Clematidis Radix	*Clematis manshurica* Ruprecht	Ranunculaceae	4	China
Lonicerae Folium	*Lonicera japonica* Thunberg	Caprifoliaceae	4	China
Phellodendri Cortex	*Phellodendrom amurense* Ruprecht	Rutaceae	3	China

### Reagents and high-performance liquid chromatography (HPLC) analysis

**Fig. 1:**
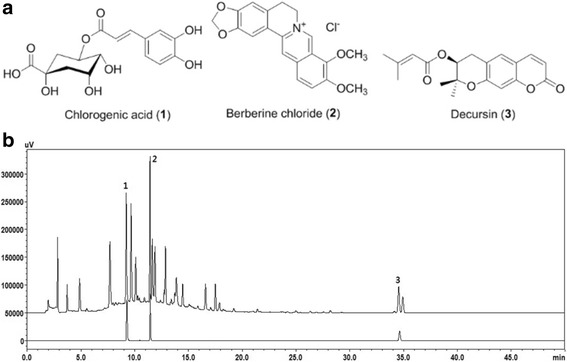
**a** Chemical structures of compounds 1–3; **b** HPLC chromatogram of a standard solution and ChondroT, with detection at 330 nm. (1) Chlorogenic acid, (2) berberine Cl, and (3) decursin

Chlorogenic acid (1) and berberine chloride (2) were purchased from U.S. Pharmacopeial Convention (Rockville, MD, USA), and Decursin (3) was purchased from ChemFaces Biochemical Co. Ltd. (Wuhan, China). The purities of the reference compounds 1–3 were 97.3%, 81.0%, and 99.4%, respectively, measured using HPLC analysis. The chemical structures of these compounds have been shown in Fig. 1a. HPLC-grade methanol, acetonitrile and water were obtained from J.T. Baker (Phillipsburg, NJ, USA), while analytical grade formic acid was purchased from Sigma–Aldrich (St. Louis, MO, USA).

For quality control of the ChondroT sample, all experiments were performed using a Shimadzu Prominence LC-20A Series (Shimadzu, Kyoto, Japan) equipped with a solvent delivery unit (LC-20AT), online degasser (DGU-20A3R), column oven (CTO-20 AC), auto-sampler (SIL-20 AC), and UV-VIS detector (SPD-20A). Data acquisition and processing were conducted using Lab solution software (version 5.73 SP3, Kyoto, Japan). Compounds 1–3 were separated using a Waters SunFire C18 column (4.6 × 250 mm; 5 μm, Milford, MA, USA) maintained at 40 °C. The mobile phases consisted of (A) 0.1% (*v*/v) aqueous formic acid and (B) 0.1% (v/v) formic acid in acetonitrile, and the gradation conditions were optimized as follows: a duration range of 0–10 min, 10%–35% B; 10–30 min, 35%–65% B; 30–35 min, 65%–80% B; 35–40 min, 80% B; 40–41 min, 80%–10% B; and 41–50 min, 10% B. The flow rate and injection volume were 1.0 mL/min and 10 μL, respectively. For HPLC analysis, lyophilized ChondroT (1 g) was dissolved in 100 mL of 70% methanol and extracted for 60 min by sonication. The extracted solution was centrifuged at 3000 rpm for 10 min, and then passed through a 0.45-μm syringe filter before HPLC analysis.

### Blood and serum tests

Blood samples were collected, and 100 μL of it was used to measure leukocytes using a Multispecies Hematology Analyzer (950, Hemavet, USA). The rest of the blood sample was used to measure aspartate aminotransferase (AST), alanine transaminase (ALT), albumin, BUN, and creatinine levels with the help of a high-speed centrifuge (VS-6000CFi, Korea) at 3000 rpm for 20 min.

### Measurement of TNF-α, IL-1β, and IL-6

TNF-α was measured using a Rat TNF-α kit (Invitrogen, USA), IL-1β was assessed using a Rat IL-1β kit (R&D Systems, USA) and IL-6 was evaluated using a Rat IL-6 kit (Invitrogen, USA). The optical densities (OD) of all samples were measured at 450 nm using Spectramax (M2, Molecular Devices, USA).

### Hematoxylin and eosin staining

The left knee joint was removed and fixed in Bouin solution for > 24 h. Decalcification was performed using a 2.5% nitric acid solution, which was changed once a day for 7 days. The removed tissue was dehydrated using a Tissue Processor (Tissue-Tek^®^ II, Japan). After deparaffinization and staining with H&E (Muto, Japan), the samples were observed under an optical microscope (Nikon, Japan).

### Safranin O-fast staining

After deparaffinization, the left knee joint was treated with Weigert’s Iron Hematoxylin solution (Sigma, USA) for 10 min and stained with 0.001% Fast Green solution (Sigma, USA) for 5 min. The sample was then incubated with 1% acetate solution for 10 s and stained with 0.1% safranin O solution (Sigma, USA) for 5 min. Thereafter, the tissue was dehydrated and observed under an optical microscope (Nikon, Japan).

### Statistical analysis

All statistical analyses were performed in SAS 9.1 version for Windows. A one-way analysis of variance was performed on each group, and the results have been expressed as mean ± standard error (SE). Comparisons between groups were performed using a post hoc least significant difference test. *p* values of < 0.05 and < 0.01 were considered statistically significant.

## Results

### Quality assessment of three marker components in ChondroT

Quality assessment of ChondroT was performed using three marker compounds and HPLC. The selected compounds were as follows: compound 1 (Lonicerae Folium), compound 2 (Phellodendri Cortex), and compound 3 (Angelicae Gigantis Radix). All analytes were separated within 40 min, and the typical chromatogram of a 70% methanol extract of ChondroT has been shown in Fig. 1b. Quantification was performed by UV-VIS detection at 330 nm based on retention time. The retention times of components 1–3 were 9.25, 11.35, and 34.51 min, respectively. Under optimized chromatography conditions, the concentrations of the marker compounds 1–3 in ChondroT were 3.67 ± 0.08, 2.41 ± 0.22, and 1.87 ± 0.18 mg/g, respectively (Table 2, Fig. 1).

**Table 2:** Concentrations of the three marker components in the ChondroT by HPLC (*n* = 3)

Compound	Mean (mg/g)	SD^a^	RSD^b^ (%)	Source
Chlorogenic acid	3.67	0.08	2.20	*Lonicera japonica*
Berberine Cl	2.41	0.22	8.99	*Phellodendron amurense*
Decursin	1.87	0.18	9.57	*Angelica gigas*

^a^ standard deviation, ^b^ relative standard deviation

### Effect of ChondroT on Proinflammatory cytokines

With regard to the effect of the amount of ChondroT administered on anti-inflammatory and inflammation-inducing agents in serum, a significant increase in the level of TNF-α was observed as compared to the intact group. The indomethacin, Joins tab, ChondroT100, and ChondroT200 groups also showed a significant increase compared with the control group.

**Fig. 2:**
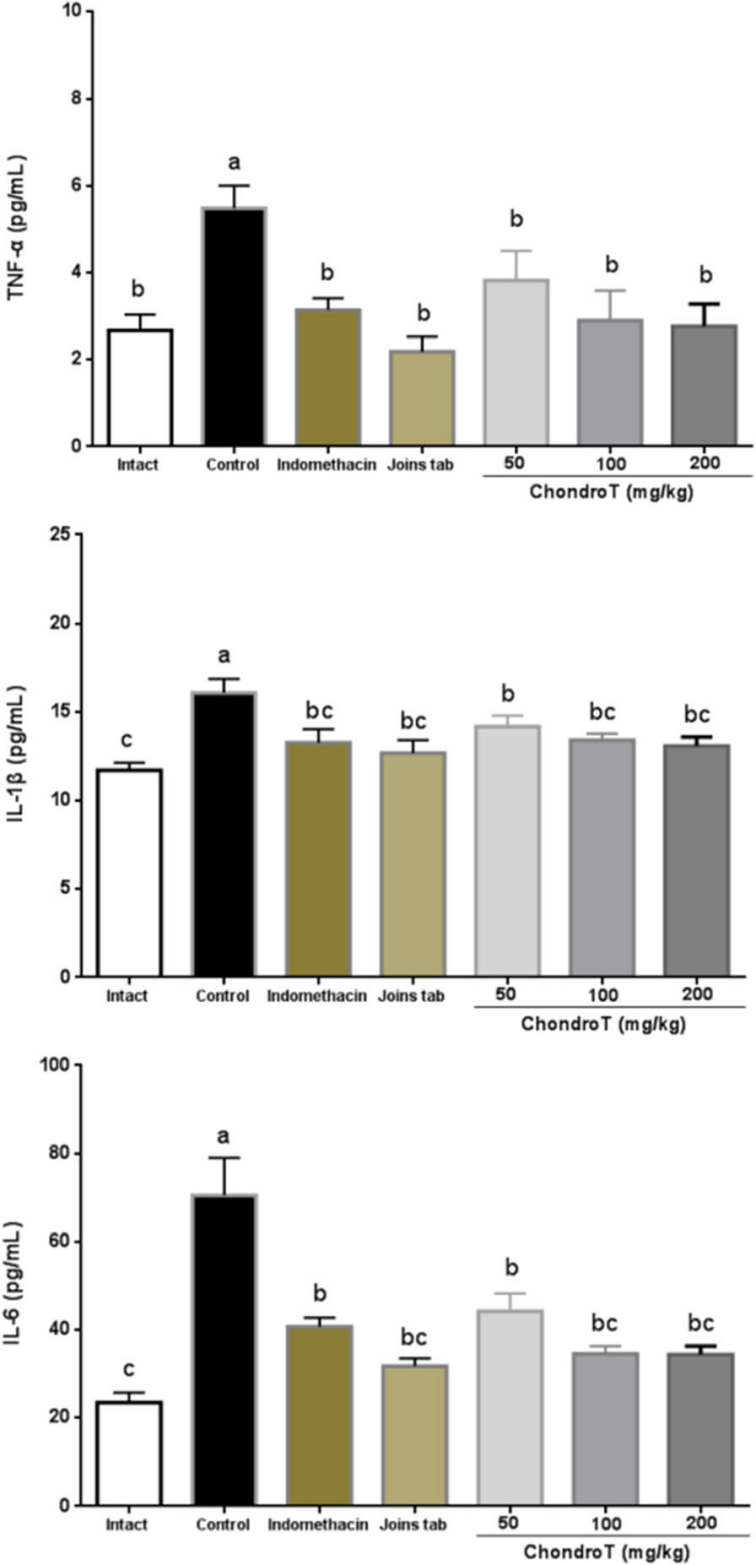
Effect of ChondroT on TNF-α, IL-1β, and IL-6 levels in rats with collagenase -induced arthritis

With regard to the level of IL-1β, the control group showed a significant increase as compared to the intact group, while the indomethacin, Joins tab, ChondroT100, and ChondroT200 groups showed a significant decrease as compared to the control group. In the case of the level of IL-6, the control group showed a significant increase as compared to the intact group, while the indomethacin, Joins tab, ChondroT50, ChondroT100, and ChondroT200 groups showed a significant decrease as compared to the control group. and in ChondroT100 and ChondroT200 groups, TNF-α, IL-1β, and IL-6 were equivalent as compared with those in the indomethacin and Joins tab (Table 3, Fig. 2).

**Table 3:** Effect of ChondroT on TNF-α, IL-1β, and IL-6 levels in rats with collagenase -induced arthritis

Groups	TNF-α (pg/mL)	IL-1β (pg/mL)	IL-6 (pg/mL)
Intact	2.67 ± 0.37^b^	11.69 ± 0.42^c^	23.47 ± 2.28^c^
Control	5.48 ± 0.43^a^	16.06 ± 0.59^a^	70.45 ± 8.52^a^
Indomethacin	3.14 ± 0.24^b^	13.24 ± 0.70^bc^	40.67 ± 1.88^b^
Joins tab	2.19 ± 0.34^b^	12.66 ± 0.73^bc^	31.74 ± 1.78^bc^
ChondroT50	3.82 ± 0.68^b^	14.17 ± 0.62^b^	44.19 ± 4.03^b^
ChondroT100	2.89 ± 0.70^b^	13.41 ± 0.35^bc^	34.58 ± 1.73^bc^
ChondroT200	2.77 ± 0.46^b^	13.07 ± 0.50^bc^	34.40 ± 2.03^bc^
F Value	4.603	4.87	10.56
*P* Value	0.0017	0.0012	0.0001

All values are mean ± standard error. Values with different superscripts within the same row are significantly different at *p* < 0.05 by LSD multiple range test

### Effect of ChondroT on histopathological changes as assessed by hematoxylin and eosin staining

**Fig. 3:**
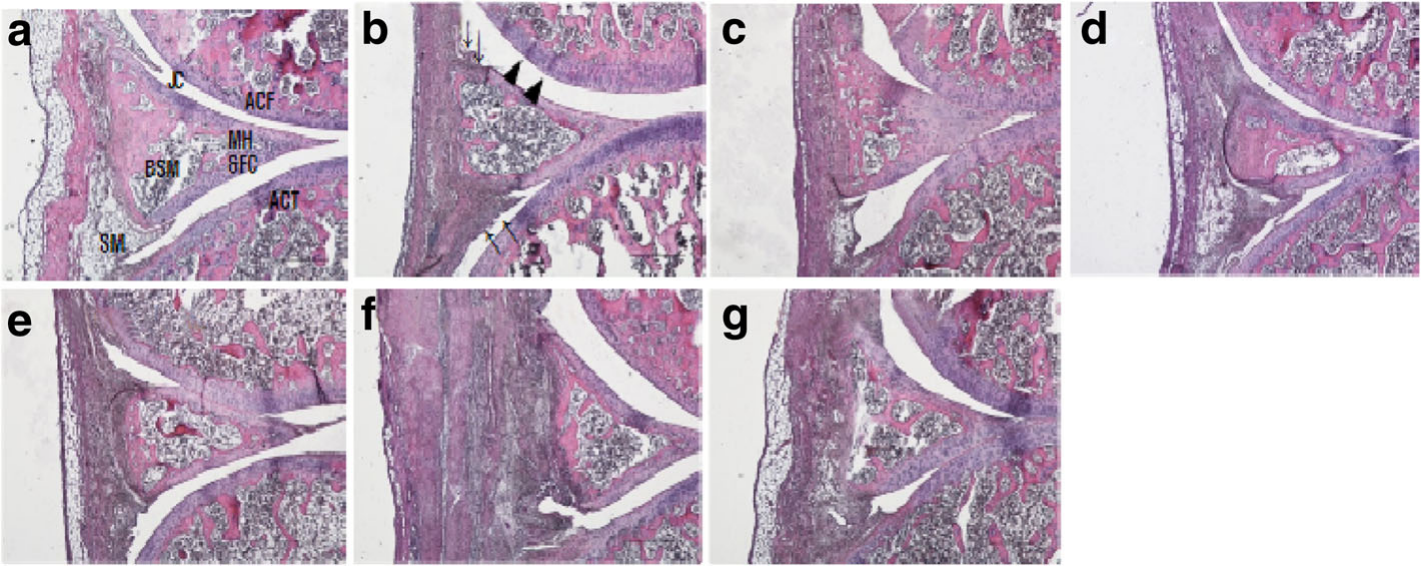
Histopathological changes (H&E stain) in the knee joint after ChondroT administration in collagenase induced arthritis rats. In H&E stain, synoviocytes, cartilage lacunae, and chondrocytes were well preserved in the ChondroT100 and ChondroT200 groups. HE-stain, Scale bars = 500 μm. **a** Intact, **b** Control, **c** Indomethacin, **d** Joins Tab, **e** ChondroT50, **f** ChondroT100, **g** ChondroT200

Histopathologically, a small portion of synovial membrane was seen to be destroyed in the control group, while the indomethacin and Joins tab groups clearly exhibited synovial membrane cells of cartilage and synovial cavity compared to the control group. In addition, the ChondroT100 and ChondroT200 groups exhibited synovial membrane cells, synovial cavity, and cartilage cells similar to the intact group (Fig. 3).

### Effect of ChondroT on histopathological changes assessed with safranin O-fast staining

**Fig. 4:**
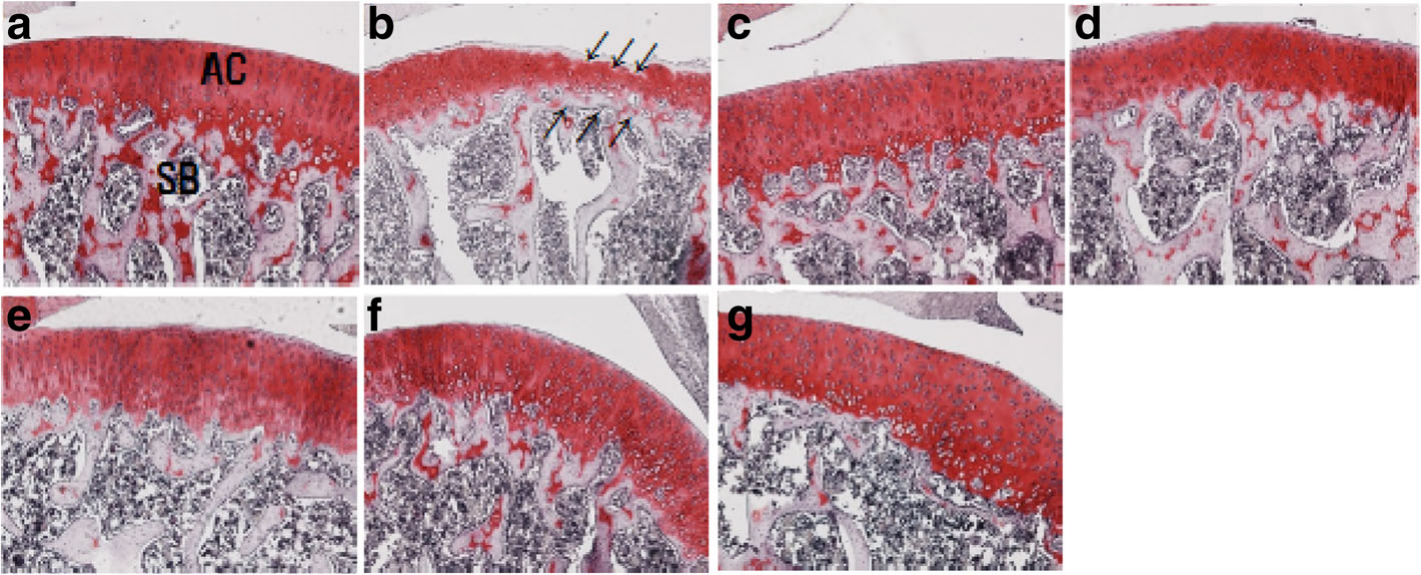
Histopathological changes (Safranin O-fast staining) in the knee joint after ChondroT administration in collagenase-induced arthritis rats. Safranin O-fast staining showed a clear reaction of proteoglycans in ChondroT200 group. Safranin O-fast stain, Scale bars = 100 μm. **a** Intact, **b** Control, **c** Indomethacin, **d** Joins Tab, **e** ChondroT50, **f** ChondroT100, **g** ChondroT200

Safranin O-fast staining showed that the positive reaction of proteoglycans in articular cartilage in the control group was lower than that of the intact group. Also, in the ChondroT200 group, the deep layer of cartilage showed a higher positive reaction for proteoglycans as compared to the control group (Fig. 4).

### Leukocyte

**Fig. 5:**
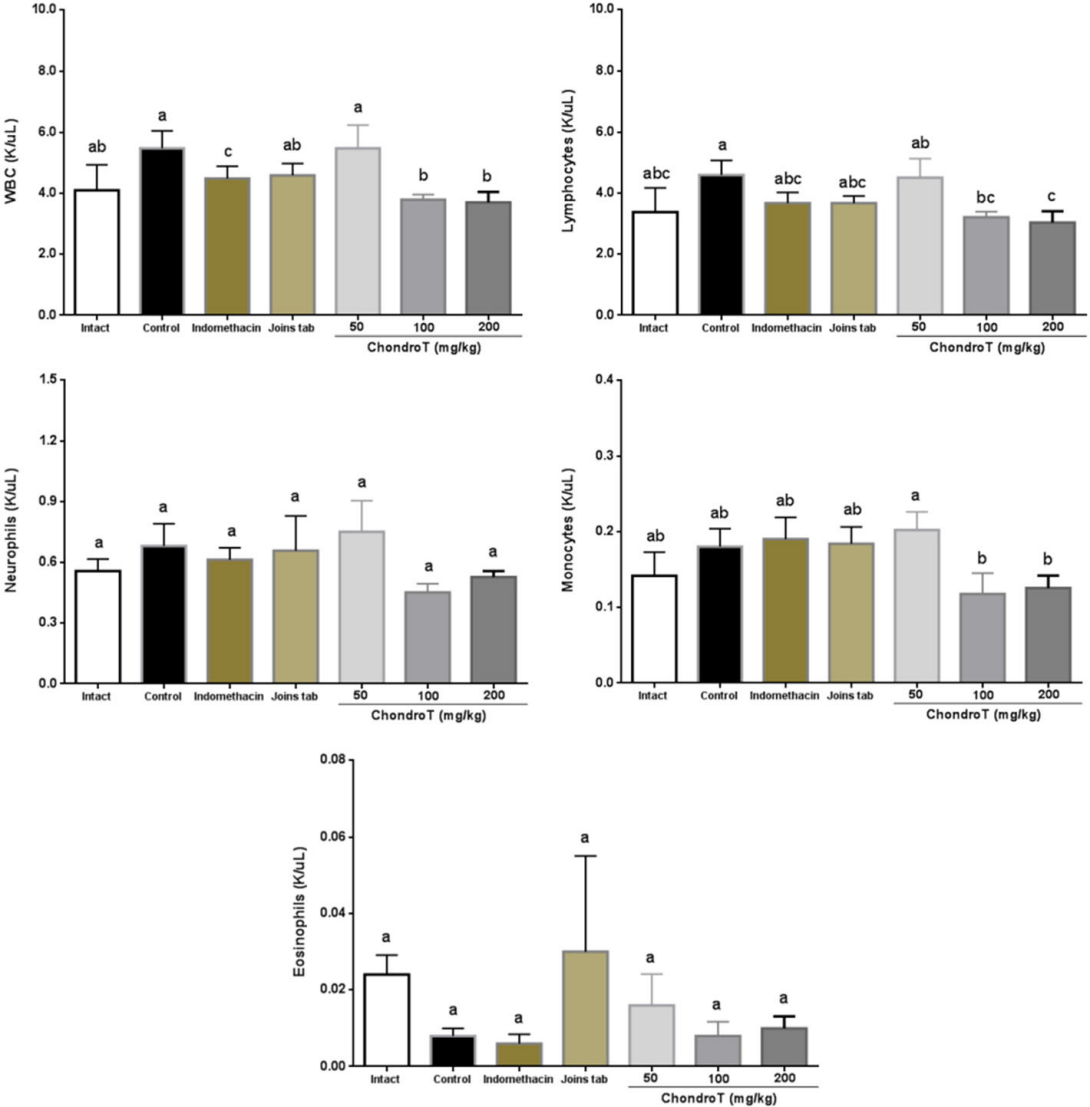
Changes in the blood WBC, neutrophils, lymphocytes, monocytes, and eosinophils contents after ChondroT administration in collagenase-induced arthritis rats

With regard to the effect of ChondroT administration on leukocyte, eosinophils in the control group exhibited a significant decrease compared with those in the intact group, whereas WBC, neutrophils, lymphocytes, and monocytes exhibited no significant differences. WBC and lymphocytes exhibited a significant decrease in ChondroT100 and ChondroT200 groups compared with those in the control group (Table 4, Fig. 5).

**Table 4:** Changes in the blood WBC, neutrophils, lymphocytes, monocytes, and eosinophils contents after ChondroT administration in collagenase-induced arthritis rats

Groups	WBC(K/μL)	NE(K/μL)	LY(K/μL)	MO(K/μL)	EO(K/μL)
Intact	4.10 ± 0.84^ab^	0.56 ± 0.06^a^	3.38 ± 0.80^abc^	0.14 ± 0.03^ab^	0.02 ± 0.01^a^
Control	5.47 ± 0.57^a^	0.68 ± 0.11^a^	4.60 ± 0.48^a^	0.18 ± 0.02^ab^	0.01 ± 0.00^a^
Indomethacin	4.49 ± 0.40c	0.61 ± 0.06^a^	3.68 ± 0.34^abc^	0.19 ± 0.03^ab^	0.01 ± 0.00^a^
Joins tab	4.59 ± 0.39^ab^	0.66 ± 0.17^a^	3.67 ± 0.24^abc^	0.18 ± 0.02^ab^	0.03 ± 0.03^a^
ChondroT50	5.48 ± 0.76^a^	0.75 ± 0.15^a^	4.51 ± 0.62^ab^	0.20 ± 0.02^a^	0.02 ± 0.01^a^
ChondroT100	3.79 ± 0.17^b^	0.45 ± 0.04^a^	3.21 ± 0.18^bc^	0.12 ± 0.03^b^	0.01 ± 0.00^a^
ChondroT200	3.70 ± 0.34^b^	0.53 ± 0.03^a^	3.04 ± 0.37^c^	0.13 ± 0.02^b^	0.01 ± 0.00^a^
F Value	1.81	0.95	1.65	1.79	0.79
*P* Value	0.1342	0.4752	0.1706	0.1368	0.5878

All values are mean ± standard error. Values with different superscripts within the same row are significantly different at *p* < 0.05 by LSD multiple range test

### Effect of ChondroT on aminotransferase

**Fig. 6:**
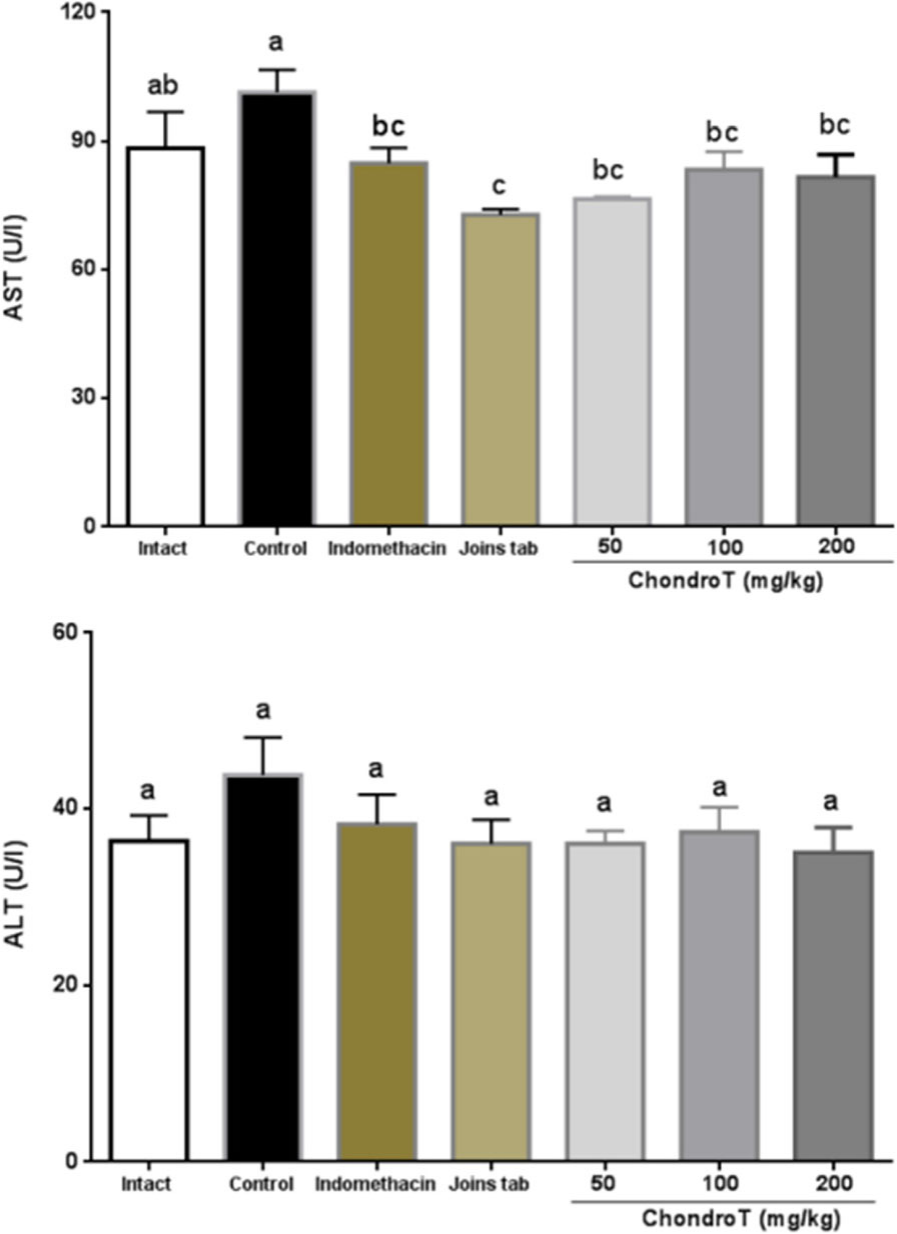
Changes in serum aminotransferase content after ChondroT administration in collagenase-induced arthritis rats

The collagenase induced control group exhibited a significant increase in AST compared to the intact group, while the indomethacin, Joins tab, ChondroT50, ChondroT100, and ChondroT200 groups showed a significant decrease compared with the control group. In the case of ALT, a decreasing tendency was observed, although this was not statistically significant (Table 5, Fig. 6).

**Table 5:** Changes in serum aminotransferase content after ChondroT administration in collagenase-induced arthritis rats

Groups	AST (U/L)	ALT (U/L)
Intact	88.3 ± 8.36^ab^	36.3 ± 2.91^a^
Control	101.2 ± 3.94^a^	43.8 ± 3.17^a^
Indomethacin	84.8 ± 3.31^bc^	38.2 ± 3.10^a^
Joins tab	72.8 ± 1.13^c^	36.0 ± 2.52^a^
ChondroT50	76.4 ± 0.62^bc^	36.0 ± 1.35^a^
ChondroT100	83.3 ± 4.20^bc^	37.3 ± 2.80^a^
ChondroT200	81.5 ± 4.77^bc^	35.0 ± 2.56^a^
F Value	3.204	0.9025
*P* value	0.0155	0.5066

All values are mean ± standard error. Values with different superscripts within the same row are significantly different at p < 0.05 by LSD multiple range test

### Effect of ChondroT on albumin, BUN, and creatinine

**Fig. 7:**
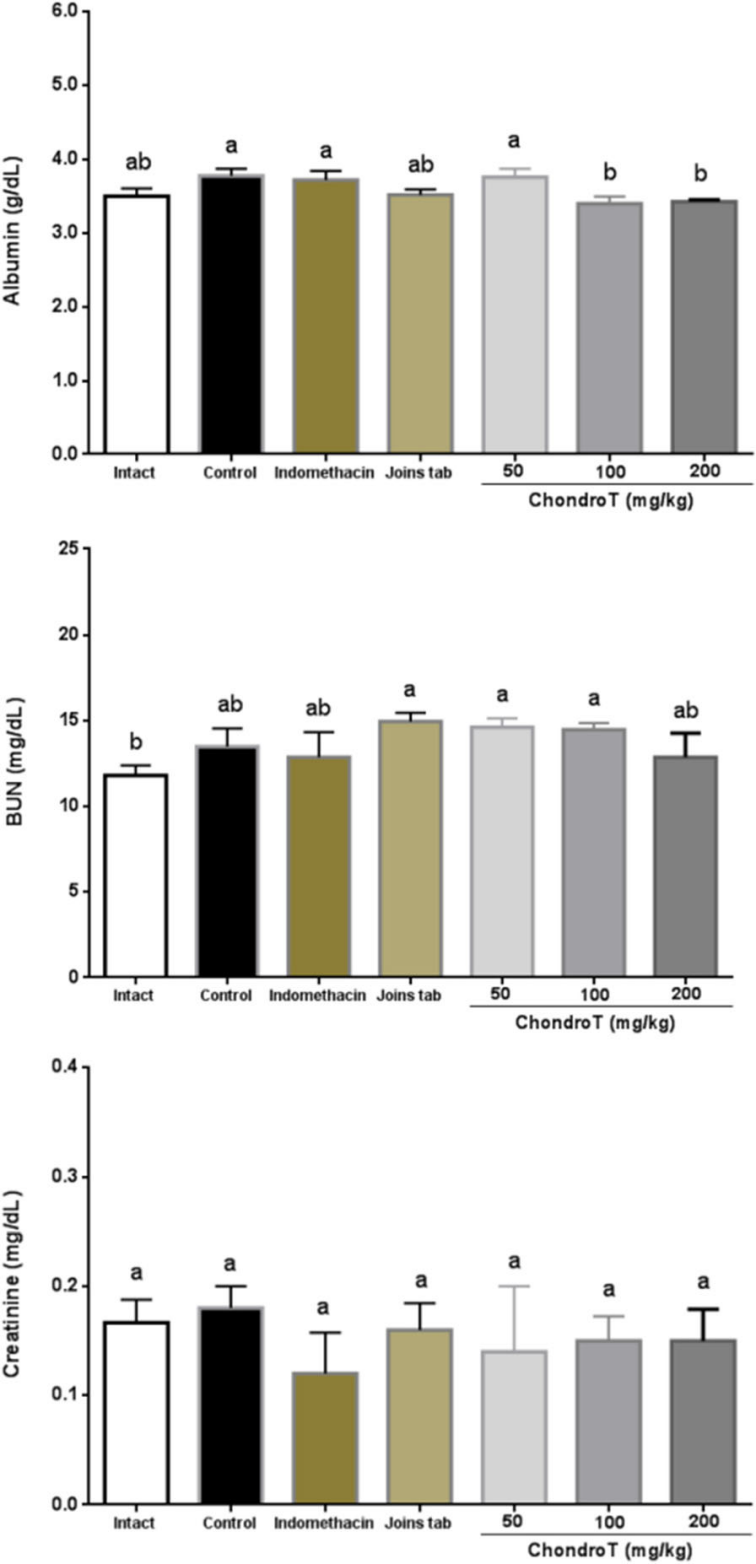
Changes in serum albumin, BUN, and creatinine content after ChondroT administration in collagenase-induced arthritis rats

The ChondroT100 and ChondroT200 groups showed a significant decrease in albumin as compared to the control group while there were no significant differences with regard to BUN and creatinine (Table 6, Fig. 7).

**Table 6:** Changes in serum albumin, BUN, and creatinine content after ChondroT administration in collagenase-induced arthritis rats

Groups	Albumin (g/dL)	BUN (mg/dL)	Creatinine (mg/dL)
Intact	3.5 ± 0.11^ab^	11.8 ± 0.59^b^	0.17 ± 0.02^a^
Control	3.8 ± 0.07^a^	13.5 ± 0.80^ab^	0.18 ± 0.01^a^
Indomethacin	3.7 ± 0.10^a^	12.9 ± 1.29^ab^	0.14 ± 0.02^a^
Joins tab	3.5 ± 0.07^ab^	14.9 ± 0.46^a^	0.13 ± 0.02^a^
ChondroT50	3.6 ± 0.05^a^	14.5 ± 0.56^a^	0.20 ± 0.03^a^
ChondroT100	3.4 ± 0.10^b^	14.5 ± 0.37^a^	0.15 ± 0.02^a^
ChondroT200	3.4 ± 0.02^b^	12.9 ± 1.26^ab^	0.15 ± 0.03^a^
F Value	2.63	1.84	0.35
*P* Value	0.0370	0.1250	0.9054

All values are mean ± standard error. Values with different superscripts within the same row are significantly different at p < 0.05 by LSD multiple range test

## Discussion

The treatment of osteoarthritis can be divided into non-pharmacologic treatment, drug treatment and surgical treatment [17]. Acetaminophen is commonly used as the primary drug along with non-steroidal anti-inflammatory drugs (NSAIDs), such as naproxen, aceclofenac, and celecoxib [18]. Besides, narcotic analgesics, such as oxymorphone, oxycodone, and oxytrex, and parenteral injections of hyaluronic acid in the joint are used selectively based on the medical team’s diagnosis. Surgical treatment is selectively used for patients with severe pain and impaired functioning [19–21].

NSAIDs are the drugs most frequently used for the treatment of pain from osteoarthritis, and are 30% more effective in eliminating pain than high dose acetaminophen [21, 22]. However, it does not have any effect on some patients, and has several side effects on the upper gastrointestinal tract including indigestion, vomiting and ulcer edema [23]. Additionally, since the action that particularly suppresses cyclooxygenase-2, also has a side effect on the cardiovascular system, its use is limited [24]. Thus, a safer and more effective medicine is required [21, 22].

Therefore, oriental medical treatment has been actively studied, as it is effective against osteoarthritis and has fewer side effects. However, most of these studies use existing prescriptions, and there are very few newly developed oriental medicines. This study verified the effects of ChondroT, a newly developed oriental medicine, on osteoarthritis using gene data processing techniques.

The indomethacin used in the positive control group is an NSAID used to treat osteoarthritis, inflammatory periodontal disease, and ankylosing spondylitis [25–28]. Joins tab is a natural drug comprising Korean virgin’s bower, *Trichosanthes kirilowii* Maxim, and Thesium and is used to treat degenerative arthritis and rheumatoid arthritis [29]. It is a newly developed drug that suppresses the destruction of cartilage and activation of joint catabolic enzymes, in addition to its simple anti-inflammatory and analgesic effects [29–31].

TNF-α is a high-level cytokine that induces catabolism, allowing generation of proteoclastic enzymes that destroy the cartilage, produced by activated monocyte and macrophagocyte, stimulating the osteoclasts gathering in the region of the topical bone resorption, leading inflammatory reaction, contributing to removing topical minerals [32]. IL-1β is known as a powerful cytokine that induces the dissolution of cartilage, produces inflammatory agents, such as prostaglandin E2 and nitric oxide, from the cartilage and synovial cells, and stimulates expression of matrix metalloproteinases [33]. It has been reported that IL-6 exhibits higher activity in the joint fluid than in the serum, suggesting an important role in osteoarthritis. It also facilitates the proliferation of synovial cells and increases the activity of osteoclasts, forming the pannus and producing proteoclastic enzymes that destroy the cartilage joint [34, 35].

As a result, in the effects of the volume of ChondroT on a change in TNF-α and IL-1β, there was no significant decrease in ChondroT50 group as compared to the control group. While there were significant decreases in ChondroT100 and ChondroT200 group, and there were significant decreases in a change in IL-6 in all ChondroT50, ChondroT100 and ChondroT200 groups as compared to the control group. so it is assumed that ChondroT has an effect on the suppression of the inflammatory reaction of osteoarthritis.

H&E and safranin O-fast staining are used for histopathological observation of synovial tissues, cartilage cells, and fibrous tissues in the knee joint. H&E staining of nucleus, proteoglycan, and protoplasm allows observation of changes in the ingredients easily as Hematoxylin is basophilic while eosin is acidophilic [36]. In safranin O-fast staining, a cationoid chromophyll is combined with an anion like keratan sulfate or chondroitin sulfate, which stains ingredients red or orange in proportion to the volume of proteoglycan distributed in the cartilage so as to allow estimation of any changes in the concentration [37, 38].

As a result of an observation after H&E staining, as compared to the control group, in ChondroT100 and ChondroT200 group, there were clear reactions of synovial cells, lacunae and cartilage cells as in the intact group, and as a result of an observation with safranin O-fast staining, there were no big differences in ChondroT50 and ChondroT100 group as compared to the control group while in ChondroT200 group, there was a higher observation of a positive reaction of proteglycans in the cartilage layer of the internal layer adjacent to the bone tissue as compared to the control group. Through the above experiment, it is noted that ChondroT200 has a positive impact on the pathological condition of the arthritis tissue.

Based on the findings of this study, it can be concluded that ChondroT suppresses inflammatory cytokines, reduces the activation of an immune response, inhibits loss of articular cartilage and protects the articular surface. Therefore, we confirmed that chondroT administration was effective for inhibiting the progression of arthritis and for protecting joints.

## Conclusions

ChondroT, a new complex herbal medication, was effective in treating a rat model of arthritis. Specifically, ChondroT significantly suppressed inflammation by a decrease in the proinflammatory cytokines, TNF-α, IL-1β, and IL-6. and it was effective in preventing articular cartilage and synovial tissue degeneration in the histophologic finding. It suggests that ChondroT, a new complex herbal medication, has therapeutic potential for the treatment of arthritis.

## Abbreviations

IL-1β: Interleukin-1β
ALT: Alanine transaminase
AST: Aspartate aminotransferase
BUN: Blood urea nitrogen
CFA: Complete Freund’s adjuvant
GHJTY: Ganghwaljetongyeum
H&E: Hematoxylin and eosin
HPLC: High-performance liquid chromatography
NSAIDs: Non-steroidal anti-inflammatory drugs
OA: Osteoarthritis
OD: Optical densities
RSD: Relative standard deviation
SD: Standard derivation
SE: Standard error
TNF-α: Tumor necrosis factor-α
